# Stimulation of poliovirus RNA synthesis and virus maturation in a HeLa cell-free in vitro translation-RNA replication system by viral protein 3CD^pro^

**DOI:** 10.1186/1743-422X-2-86

**Published:** 2005-11-21

**Authors:** David Franco, Harsh B Pathak, Craig E Cameron, Bart Rombaut, Eckard Wimmer, Aniko V Paul

**Affiliations:** 1 Department of Molecular Genetics and Microbiology, School of Medicine, Stony Brook University, Stony Brook, N. Y. 11790, USA; 2 Department of Biochemistry and Molecular Biology, Pennsylvania State University, University Park, Pennsylvania 16802, USA; 3 Department of Microbiology and Hygiene, Vrije Universiteit Brussel, B-1090 Brussels, Belgium

**Keywords:** Poliovirus, RNA replication, virus maturation, HeLa cell-free translation-RNA replication system

## Abstract

Poliovirus protein 3CD^pro ^possesses both proteinase and RNA binding activities, which are located in the 3C^pro ^domain of the protein. The RNA polymerase (3D^pol^) domain of 3CD^pro ^modulates these activities of the protein. We have recently shown that the level of 3CD^pro ^in HeLa cell-free in vitro translation-RNA replication reactions is suboptimal for efficient virus production. However, the addition of either 3CD^pro ^mRNA or of purified 3CD^pro ^protein to in vitro reactions, programmed with viral RNA, results in a 100-fold increase in virus yield. Mutational analyses of 3CD^pro ^indicated that RNA binding by the 3C^pro ^domain and the integrity of interface I in the 3D^pol ^domain of the protein are both required for function. The aim of these studies was to determine the exact step or steps at which 3CD^pro ^enhances virus yield and to determine the mechanism by which this occurs. Our results suggest that the addition of extra 3CD^pro ^to in vitro translation RNA-replication reactions results in a mild enhancement of both minus and plus strand RNA synthesis. By examining the viral particles formed in the in vitro reactions on sucrose gradients we determined that 3CD^pro ^has only a slight stimulating effect on the synthesis of capsid precursors but it strikingly enhances the maturation of virus particles. Both the stimulation of RNA synthesis and the maturation of the virus particles are dependent on the presence of an intact RNA binding site within the 3C^pro ^domain of 3CD^pro^. In addition, the integrity of interface I in the 3D^pol ^domain of 3CD^pro ^is required for efficient production of mature virus. Surprisingly, plus strand RNA synthesis and virus production in in vitro reactions, programmed with full-length transcript RNA, are not enhanced by the addition of extra 3CD^pro^. Our results indicate that the stimulation of RNA synthesis and virus maturation by 3CD^pro ^in vitro is dependent on the presence of a VPg-linked RNA template.

## Introduction

The HeLa cell-free in vitro translation-RNA replication system [1] offers a novel and useful method for studies of the individual steps in the life cycle of poliovirus. These processes include the translation of the input RNA, processing of the polyprotein, formation of membranous replication complexes, uridylylation of the terminal protein VPg, synthesis of minus and plus strand RNA, and encapsidation of the progeny RNA genomes to yield authentic progeny virions [1-4]. Although these processes occurring in vitro represent, in large part, what happens in virus-infected cells, there are also differences between virus production in vivo and in vitro. In the in vitro system a large amount of viral RNA (~1 × 10^11 ^RNA molecules) has to be used, as template for translation and replication, in order to obtain infectious viral particles and the yield of virus is still relatively low. This has been attributed to insufficient concentrations of viral proteins for RNA synthesis or encapsidation, to differences in membranous structures or the instability of viral particles in vitro [3,5]. With the large amount of input RNA the level of translation in vitro is relatively high from the beginning of incubation and hence complementation between viral proteins is more efficient than in vivo [6,7]. We have recently observed that in vitro translation-RNA replication reactions, programmed with viral RNA, contain suboptimal concentrations of the important viral precursor protein 3CD^pro ^for efficient virus production. By supplying the in vitro reactions at the beginning of incubation either with 3CD^pro ^mRNA or purified 3CD^pro ^protein the virus yield could be enhanced 100 fold [8,9]. Our results also indicated that both the 3C^pro ^proteinase and 3D^pol ^polymerase domains of the protein are required for its enhancing activity.

**Figure 1:**
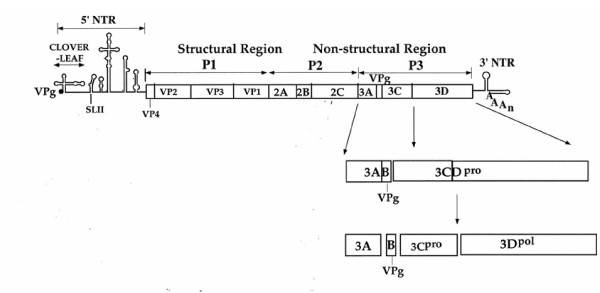
Genomic structure of poliovirus and processing of the P3 domain of the polyprotein. The plus strand RNA genome of poliovirus is illustrated with the terminal protein VPg covalently linked to the 5' end of the RNA. The 5' nontranslated region (NTR) and 3' NTR are shown with single lines. The genome is terminated with a poly(A) tail. The polyprotein (open box) contains structural (P1) and nonstructural (P2 and P3 domains) that are processed into precursor and mature proteins. Processing of the P3 domain by 3C^pro^/3CD^pro ^is shown enlarged.

Poliovirus (PV), a member of the *Picornaviridae *virus family, replicates its plus strand genomic RNA within replication complexes contained in the cytoplasm of the infected cell. These complexes provide a suitable environment for increased local concentration of all the viral and cellular proteins needed for RNA replication and encapsidation of the progeny RNA genomes. Translation of the incoming plus strand RNA genome of PV yields a polyprotein, which is cleaved into functional precursors and mature structural and nonstructural proteins (Fig. 1). This is followed by the synthesis of a complementary minus strand RNA, which is used as template for the production of the progeny plus strands [reviewed in [10]]. Although the process of viral particle assembly is not fully understood it is believed to occur by the following pathway: The P1 precursor of the structural proteins is cleaved into VP0, VP1 and VP3, which form a noncovalent complex, the protomer [11]. The protomers associate into pentamers and six pentamers form an icosahedral particle (empty capsid) enclosing the progeny plus strand RNA yielding provirions. It is unclear whether the progeny RNA is inserted into the empty capsid or whether the pentamers condense around the RNA [12,13]. Maturation is completed by the cleavage of VP0 into VP2 and VP4, possibly by a RNA-dependent autocatalytic mechanism [11]. From the nonstructural viral proteins 2C^ATPase ^[14] and VPg [15] have been proposed to have a role in encapsidation but their functions are not yet known.

The viral proteins most directly involved in RNA replication include protein 3AB, the precursor of 3A, which is a small membrane binding and RNA binding protein, the terminal protein VPg, RNA polymerase 3D^pol ^and proteinase 3C^pro^/3CD^pro^. As a proteinase 3CD^pro ^is responsible for the processing of the capsid precursor [16] but it also has very important functions as an RNA binding protein [17-21]. It forms complexes with the 5' cloverleaf structure in PV RNA either in the presence of cellular protein PCBP2 [18,22] or viral protein 3AB [19]. The interaction between PCBP2, 3CD^pro ^and the cloverleaf has been proposed to mediate the switch from translation to RNA replication [23] and the circularization of PV RNA through interaction with poly(A) binding protein bound to the poly(A) tail of the genome [24]. In addition, 3CD^pro ^binds to the *cre*(2C) element [20,21], and to the 3'NTR in a complex with 3AB [19]. Polypeptide 3CD^pro ^is also a precursor of proteinase 3C^pro ^and RNA polymerase 3D^pol^. The 3C^pro ^domain of the polypetide contains both the proteinase active site and the primary RNA binding domain [25,26]. The function of the 3D^pol ^domain appears to be to modulate these activities of the protein [27,28] and it also contains RNA binding determinants [27]. By itself 3D^pol ^is the RNA dependent RNA polymerase, which possesses two distinct synthetic activities. It elongates oligonucleotide primers on a suitable template [29] and it links UMP to the hydroxyl group of a tyrosine in the terminal protein VPg [20]. The 3D^pol ^polypeptide possesses a structure similar to other nucleic acid polymerases of a right hand with palm, thumb and finger subdomains [30]. Interaction between polymerase molecules along interface I results in a head to tail oligomerization of the protein, which is important for its biological functions [31].

The aim of these studies was to determine how the addition of extra 3CD^pro ^protein to in vitro translation RNA-replication reactions, programmed with viral RNA, stimulates virus synthesis by 100 fold. In the presence of extra 3CD^pro ^we have observed a mild stimulation of both minus and plus strand RNA synthesis. The primary effect of 3CD^pro^, however, is the enhancement of virus maturation resulting in a striking increase in the specific infectivity of the virus particles produced. Both of these processes are dependent on the RNA binding activity of the protein in the 3C^pro ^domain. Mutational analysis of 3CD^pro ^suggests that the formation of 155S mature virions also requires an intact interface I in the 3D^pol ^domain of the protein. Interestingly, plus strand RNA synthesis and virus production in translation RNA-replication reactions, programmed with PV transcript RNA, are not stimulated by 3CD^pro^.

## Results

### Effect of 3CD^pro^(3C^pro^H40A) on viral RNA synthesis in in vitro translation-RNA replication reactions

**Figure 2:**
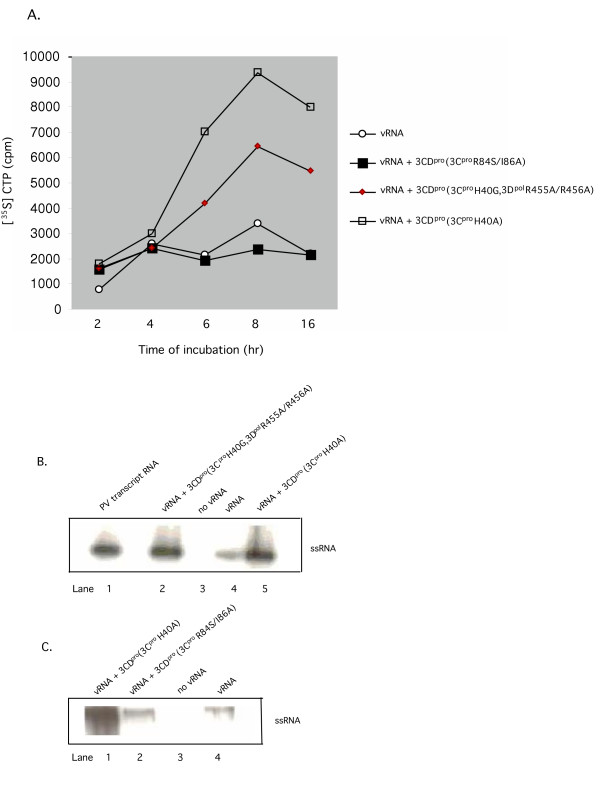
Effect of 3CD^pro^(3C^pro^H40A) on viral RNA synthesis in the translation-RNA replication system. (A) Comparison of the stimulating activities of purified 3CD^pro^(3C^pro^H40A) with mutant 3CD^pro^(3C^pro^R84S/I86A) or 3CD^pro^(3C^pro^H40G; 3D^pol^R455A/R456A) on total viral RNA synthesis. Translation-RNA replication reactions were carried out in the presence of [α-^35^S]CTP. Where indicated purified 3CD^pro ^proteins (5.5 nM) or mRNA (1.4 μg/ml) was added at t = 0 hr. Samples were taken at the indicated time points (Method I) and the total amount of label incorporated into polymer was determined with a filter-binding assay, as described in Materials and Methods. (B), (C) Comparison of the stimulating activities of purified 3CD^pro^(3C^pro^H40A) with that of mutants 3CD^pro^(3C^pro^H40G, 3D^pol^R455A/R456A) and 3CD^pro^(3C^pro^R84S/I86A), respectively, on plus strand RNA synthesis. Translation-RNA replication reactions were carried out for 4 hr and the replication complexes were isolated by centrifugation (Materials and Methods). The pellets were resuspended in translation reactions lacking viral RNA in the presence of [α-^32^P]CTP and the samples were incubated for 1 hr at 34°C. Following extraction and purification the RNA products were applied to a nondenaturing agarose gel (Materials and Methods). A [^32^P]UMP-labeled PV transcript RNA was used as a size marker for full length PV RNA.

We have previously shown that translation of 3CD^pro ^mRNA along with the viral RNA template in in vitro translation-RNA replication reactions, programmed with viral RNA, enhances total RNA synthesis about 3 fold [9]. The addition of 3CD^pro^, however, had no effect on the translation of the input viral RNA or processing of the polyprotein [8,9]. We have now extended these results by testing the effect of mutations in 3CD^pro ^on the ability of the protein to stimulate RNA synthesis. Translation-RNA replication reactions were incubated at 34°C either in the absence or presence of extra purified 3CD^pro^(3C^pro^H40A). This protein, which contains a proteinase active site mutation, H40A, served as the positive control in all of our experiments. Samples were taken at 2-hour intervals and these were incubated with [α-^35^S]CTP for 1 hour. RNA synthesis was measured by the incorporation of label into polymer using a filter-binding assay. As shown in Fig. 2A, RNA synthesis is maximal 8 hrs after the start of translation and by 16 hr the total amount of RNA present in the reaction decreases. At the peak of RNA synthesis there is a 3-fold difference between reactions containing extra 3CD^pro^(3C^pro^H40A) and those to which no additional protein has been added.

Protein 3CD^pro ^is the precursor of both proteinase 3C^pro ^and polymerase 3D^pol^. The 3C^pro ^domain contains both the proteinase and the RNA binding site [25,26]. While the primary RNA binding determinant of 3CD^pro ^lies in 3C^pro^, lower affinity binding determinants are located in the 3D^pol ^domain [27,28]. We have recently shown that a mutation (3C^pro^R84A/I86A) in the RNA binding domain of 3CD^pro ^abolishes that ability of the protein to stimulate virus production in the in vitro system [8]. To examine the effect of these mutations on RNA synthesis we have carried out translation-RNA replication reactions in the presence 3CD^pro^(3C^pro^R84S/I86A) mRNA. As shown in Fig. 2A, the mutation totally abolished the stimulatory activity of 3CD^pro^(3C^pro^H40A) in RNA synthesis suggesting that RNA binding is required for participation of the extra 3CD^pro^(3C^pro^H40A) in genome replication.

Our previous results indicated that the 3D^pol ^domain of 3CD^pro ^is also required for the ability of 3CD^pro ^to stimulate virus synthesis in the in vitro system [8]. This conclusion was based on the observation that two groups of mutations R455A/R456A [32] and D339A/S341A/D349A [33] in the 3D^pol ^domain of the protein abolished the enhancement of virus yield in the in vitro system [8]. These complementary mutations in the thumb and palm subdomains of the protein, respectively, are located at interface I of the 3D^pol ^protein structure and have been found to disrupt the oligomerization of the polypeptide [32,33]. Previous studies have indicated that oligomeric forms of the 3D^pol ^polypeptide are required for enzyme function [31]. To determine the effect of 3CD^pro^(3C^pro^H40G, 3D^pol^R455A/R456A) on RNA synthesis we added the purified mutant protein to translation RNA-replication reactions. This mutant protein exhibited a 2-fold stimulation in RNA synthesis, only slightly lower than what is obtained with 3CD^pro^(3C^pro^H40A) (Fig. 2A). This result indicates that 3D^pol ^residues R455 and R456 are not important for the stimulatory activity of 3CD^pro ^in RNA synthesis. The effect of the other mutant 3CD^pro ^protein (3D^pol^D339A/S341A/D349A) on RNA synthesis was not analyzed.

### 3CD^pro^(3C^pro^H40A) has a small stimulatory effect on both minus and plus strand RNA synthesis

To examine the effect of 3CD^pro ^on plus strand RNA synthesis we translated the viral RNA for 4 hr in the absence or presence of extra 3CD^pro^(3C^pro^H40A). The initiation complexes [34] were isolated by centrifugation and resuspended in reaction mixtures lacking viral RNA but containing [α-^32^P]CTP. After 1 hr of incubation the RNA products were applied to a nondenaturing agarose gel together with a [α-^32^P]-labeled full-length poliovirus RNA transcript as a size marker (Fig. 2B, lane 1). The yield of plus strand RNA product obtained from these reactions was equally enhanced by the addition of extra 3CD^pro^(3C^pro^H40A) or by mutant 3CD^pro^(3C^pro^H40G, 3D^pol^R455A/R456A) protein (Fig. 2B, compare lane 4 with lanes 2 and 5). No product was formed in the absence of a viral RNA template (Figs. 2B and 2C, lane 3). When 3CD^pro ^mRNA, containing the R84S/I86A mutations in the RNA binding domain of 3C^pro^, was cotranslated with the input viral RNA no stimulation of plus strand RNA synthesis was observed (Fig. 2C, compare lanes 2 and 4). These results indicate that RNA binding by the extra 3CD^pro^(3C^pro^H40A) is required for the stimulation of plus strand RNA synthesis but mutation R455A/R456A in the 3D^pol ^domain of the protein is not important for this process.

**Figure 3:**
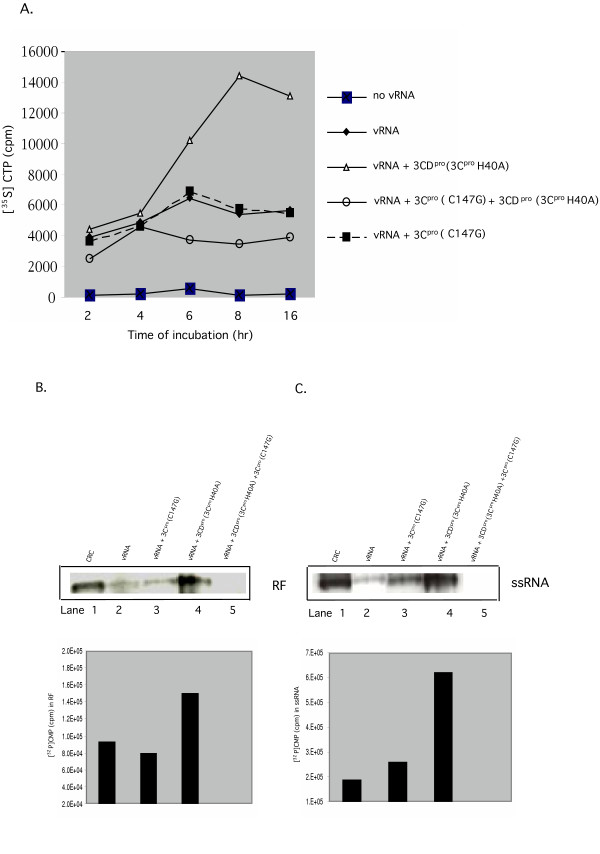
Inhibition of 3CD^pro^(3C^pro^H40A)-stimulated RNA synthesis by 3C^pro^(C147G) in vitro. (A) Inhibition of 3CD^pro^(3C^pro^H40A)-stimulated total viral RNA synthesis by 3C^pro^(C147G). Translation-RNA replication reactions were incubated for the indicated time periods in the presence of [α-^35^S]CTP (Method II) either in the absence or presence of 3CD^pro^(C^pro^H40A) (5.5 nM). The total amount of label incorporated into polymer was determined with a filter-binding assay, as described in Materials and Methods. Where indicated 3C^pro^(C147G) was added to the reactions at t = 0 either alone or together with 3CD^pro^(3C^pro^H40A). (B), (C) Inhibition of 3CD^pro^(3C^pro^H40A)-stimulated minus (B) and plus strand (C) RNA synthesis by 3C^pro^(C147G). Translation-RNA replication reactions were carried out in the presence of guanidine HCl for 4 hr and the replication complexes were isolated by centrifugation (Materials and Methods). The pellets were resuspended in translation reactions lacking viral RNA in the presence of [α-^32^P]CTP and the samples were incubated for 1 hr at 34°C. Following extraction and purification of the RNAs the samples were analyzed on a nondenaturing agarose gel (Materials and Methods). RF: double stranded replicative form RNA; ssRNA: single stranded RNA; CRC: [^32^P]-labeled RNA products from crude replication complexes (Materials and Methods).

To compare the stimulatory effect of 3CD^pro^(3C^pro^H40A) on both minus and plus strand RNA synthesis we used preinintiation replication complexes [2,34], which were collected after 4 hr of incubation of the reactions in the presence of 2 mM guanidine HCl, a potent inhibitor of poliovirus RNA replication. The complexes were resuspended in reactions lacking viral RNA and guanidine and were incubated for an hour with [α-^32^P]CTP. The RNA products were resolved on a nondenaturing agarose gel. Minus strand RNA synthesis was estimated from the amount of replicative form (RF), in which the minus strand is hybridized to the plus strand template RNA. As shown in Fig. 3B, minus and plus strand RNA synthesis are enhanced about 2-fold and 3-fold, respectively, when the reactions contain extra 3CD^pro^(3C^pro^H40A). Poliovirus RF and ssRNA obtained from a reaction in which HeLa extracts were replaced by crude replication complexes (CRCs), isolated from PV-infected HeLa cells [35], were used as a size marker for the RF and the plus strand RNA (ssRNA) (Figs. 3B, and 3C, lane 1).

### The addition of 3CD^pro^(3C^pro^H40A) and 3C^pro^(C147G) together totally blocks RNA synthesis in translation-RNA replication reactions

We have recently shown that purified 3C^pro^(C147G) protein, containing a proteinase active site mutation, when added alone to in vitro translation-RNA replication reactions, has no effect on virus yield. However, when included in reactions along with extra 3CD^pro^(3C^pro^H40A) the production of virus is reduced about 1 × 10^4 ^fold [8]. To determine whether the inhibitory effect of 3C^pro^(C147G) is at the level of RNA synthesis, we have examined the time course of RNA synthesis in the presence of both proteins by measuring the amount of [α-^35^S]UMP incorporated into polymer. As shown in Fig. 3A, the effect of these proteins on RNA synthesis fully parallels their effect on virus synthesis [8]. 3CD^pro^(3C^pro^H40A) stimulates RNA synthesis up to 3-fold while 3C^pro^(C147G) alone exhibits no significant enhancement of the RNA yield. When the two proteins are added together there is essentially no increase in the total amount of RNA produced over a period of 16 hours. Control reactions, lacking a viral RNA template exhibited very little, if any, incorporation of label into a polymeric product (Fig. 3A). All other samples showed some incorporation of label into polymer, over what is measured in the absence of viral RNA (Fig. 3A). This is most likely a result of end labeling of the input viral RNA by newly translated 3D^pol ^or priming by traces of degraded RNA.

To determine whether 3C^pro^(C147G) inhibits plus or minus strand RNA synthesis we labeled with [α-^32^P]CMP the RNA products formed in preinintiation replication complexes during a 1 hr incubation period, as described above. The samples were analyzed on a nondenaturing agarose gel and as a size marker we used [α-^32^P]CMP-labeled RNA products made in CRCs (Figs. 3B and 3C, lane 1). Two kinds of products were visible on the gel, the newly made single stranded RNA (ssRNA) and the double stranded replicative intermediate (RF). As shown on Fig. 3B, 3C^pro^(C147G) alone has very little, if any, effect on the yield of either of the 2 kinds of RNA products (Fig. 3B and 3C, compare lanes 2 and 3). In the presence of both 3C^pro^(C147G) and 3CD^pro^(3C^pro^H40A), however, the synthesis of both products is completely inhibited (Figs. 3B and 3C, compare lane 4 and lane 5).

### 3CD^pro^(3C^pro^H40A) has a small stimulating effect on the early steps of viral particle assembly

**Figure 4:**
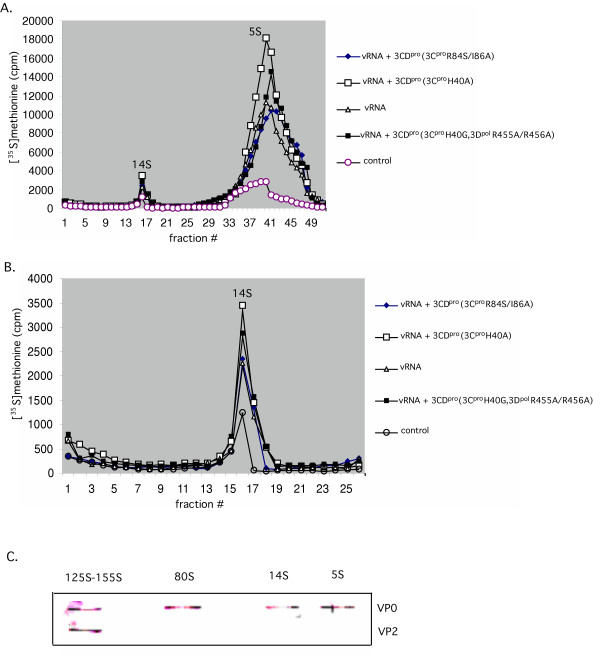
Effect of 3CD^pro^(3C^pro^H40A) on the early stages of poliovirus assembly in vitro. Translation-RNA replication reactions were carried out in the presence of [^35^S]TransLabel, as described in Materials and Methods. When indicated purified 3CD^pro^(3C^pro^H40A) protein (5.5 nM) or mRNA (1.4 μg/ml) was added to the reactions at t = 0 hr and the samples were incubated for 16 hr at 34°C. Following RNase treatment and dialysis the samples were loaded on a 5–20% sucrose gradient (Materials and Methods). The samples were centrifuged for 15 hr at 40,000 RPM in a SW41 rotor at 4°C for the separation of 5S protomers and 14S pentamers. The amount of radioactivity at the bottom of the tubes of the gradients was not determined. (A) Comparison of samples obtained in the absence or presence of 3CD^pro^(3C^pro^H40A) and mutant 3CD^pro ^protein 3D^pol^(H40G, R455A/R456A) or mRNA 3C^pro^(R84S/I86A). (B) The 14S peak from section (A) is shown enlarged; (C) Western blot analysis with anti VP2 antibodies of samples from the 5S and 14S peaks from the gradient shown on Fig. 4A. The same analysis of the 80S and 155S peaks from the gradient shown on Fig. 5.

**Figure 5:**
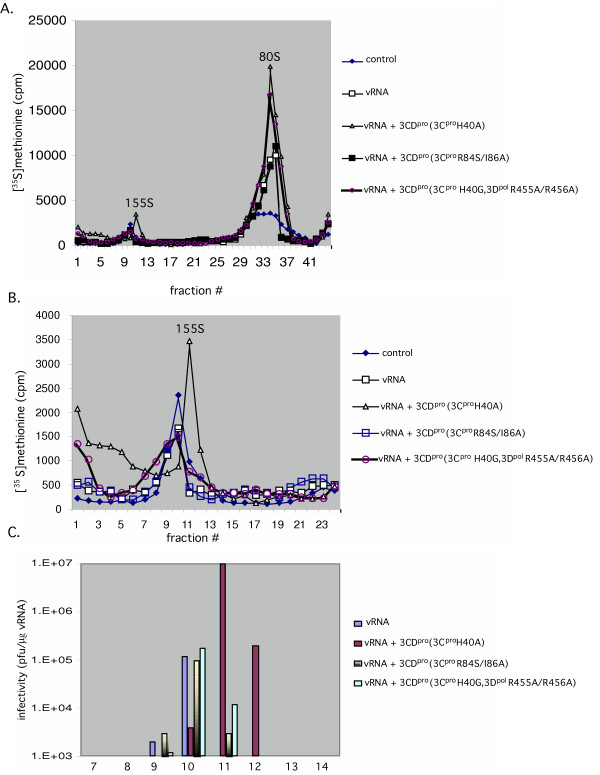
Effect of 3CD^pro^(3C^pro^H40A) on the late stages of poliovirus assembly in vitro. Translation-RNA replication reactions were carried out in the presence of [^35^S]TransLabel, as described in Materials and Methods. When indicated purified 3CD^pro^(3C^pro^H40A) protein (5.5 nM) or mRNA (1.4 μg/ml) was added to the reactions at t = 0 hr and the samples were incubated for 16 hr at 34°C. As a control, poliovirus proteins labeled with [^35^S]TransLabel in vivo in HeLa cells, were used. Following RNase treatment and dialysis the samples were loaded on a 5–20% sucrose gradient (Materials and Methods). The samples were centrifuged for 80 min at 40,000 RPM in a SW41 rotor at 4°C for the separation of 80S empty capsids and 155S virus particles (provirions and virions). (A) Comparison of samples obtained in the absence or presence of 3CD^pro^(3C^pro^H40A) and mutant 3CD^pro ^protein 3D^pol^(H40G, R455A/R456A) or mRNA 3C^pro^(R84A/I86A). (B) The 155S peak from section (A) is shown enlarged. (C) Plaque assays of fractions 7–14 in the 155S peak.

The data shown before indicated a modest increase in viral RNA synthesis in the presence of extra 3CD^pro^(3C^pro^H40A) whereas the production of infectious virus was stimulated about 100 fold. The fact that there is such a large discrepancy between the extent of stimulation of RNA synthesis and virus production by 3CD^pro^(3C^pro^H40A) suggested to us the possibility that this protein has an additional role at a subsequent step in the viral life cycle, the encapsidation of the progeny viral RNAs. To examine at which step of assembly this might occur, we labeled the viral proteins with [^35^S]-methionine in the in vitro reactions and analyzed the viral particles produced after 15 hr incubation either in the absence or presence of 3CD^pro^(3C^pro^H40A). The samples were first loaded on a 5–20% sucrose gradient and sedimented for 15 hr, which resulted in the separation of the 5S protomers and 14S pentamers from the large capsid precursors and mature virions [36]. As a size marker for these small capsid precursors, a parallel gradient was run, onto which a sample of [^35^S]-labeled PV-infected HeLa cell lysate was applied (designated as control in Figs. 4 and 5). The amount of the 5S and 14S precursors is enhanced less than two fold by the presence of extra 3CD^pro^(3C^pro^H40A) in the reactions (Figs. 4A and 4B). Similarly, reactions supplemented with mutant 3CD^pro ^proteins, containing mutations either at the RNA binding site of 3C^pro^(R84A/I86A) or at interface I in 3D^pol^(R455A/R456A), exhibited very little increase in the total amount of 5S and 14S particles, when compared to reactions lacking 3CD^pro^(3C^pro^H40A) (Figs. 4A and 4B).

To confirm the presence of uncleaved VP0 in the 5S and 14S peak fractions of the gradient derived from reactions supplemented with extra 3CD^pro^(3C^pro^H40A), we used Western blot analyses with anti VP2 polyclonal antibody (Fig. 4C). As expected, only VP0 and no VP2 could be detected in the 5S and 14S peak fractions containing these small capsid precursors (Fig. 4C).

### 3CD^pro^(3C^pro^H40A) has a small stimulatory effect on the late stages of particle assembly

In the next set of experiments we examined the effect of 3CD^pro^(3C^pro^H40A) on the formation of 80S (empty capsids) and 155S particles (provirion and mature virus). As we discussed before, the role of the 80S particle in viral assembly is unclear. The experimental evidence available at this time favors the hypothesis that empty capsids are dead-end products rather than true intermediates of particle assembly [12,13]. The particle thought to be the direct precursor of the mature virus is the provirion, a structure containing 60 copies of VP0, VP1 and VP3 and the viral RNA [37]. The difference between provirions and mature virus is that in the latter the particle is stabilized by the cleavage of VP0 to VP2 and VP4.

**Figure 6:**
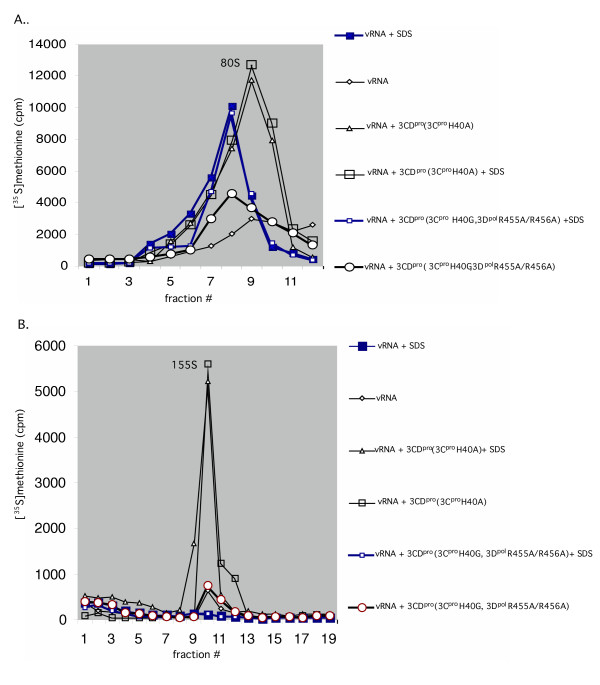
3CD^pro^(3C^pro^H40A) enhances the specific infectivity of virus particles produced in vitro. Translation-RNA replication r*eac*tions were carried out in the presence of [^35^S]TransLabel, as described in Materials and Methods. Where indicated purified 3CD^pro^(3C^pro^H40A) or 3CD^pro^(3C^pro^H40G, 3D^pol^R455A/R456A) protein (5.5 nM) was added to the reactions at t = 0 hr and the samples were incubated for 16 hr at 34°C. Following RNase treatment and dialysis, 0.1% of SDS was added to the samples, as indicated. They were loaded on a 5–20% sucrose gradient (Materials and Methods) and centrifuged for 80 min at 40,000 RPM in a SW41 rotor at 4°C. (A) the 80S peak is shown; (B) the 155S peak is shown.

The 80S and 155S viral particles, labeled with [^35^S]-methionine in vitro, were separated by sedimentation in a 5–20% sucrose gradient for 80 min. Under our experimental conditions the provirions (125S) and mature virus (155S) comigrate [36,37]. As shown in Fig. 5A the yield of 80S particles is stimulated about 2 fold by 3CD^pro^(3C^pro^H40A) and by 3CD^pro^(3C^pro^H40G, 3D^pol^R455A/R456A) but not by 3CD^pro^(3C^pro ^R84S/I86A). The formation of 155S particles is enhanced about 3–7 fold by 3CD^pro^(3C^pro^H40A) but not by the 3CD^pro ^proteins that contain the 3D^pol^R455A/R456A or 3C^pro ^R84S/I86A mutations (Figs 5A,5B, 6). To confirm the presence of mature virions in the 155S peak fractions, derived from reactions supplemented with extra 3CD^pro^(3C^pro^H40A), we used Western blot analysis with anti VP2 polyclonal antibody. As expected, both VP2 and VP0 were observed in the 155S peak but only VP0 was present in the 80S peak fractions of the gradient (Fig. 4C).

### 3CD^pro^(3C^pro^H40A) strongly enhances the production of mature viral particles

As we discussed above, the extra 3CD^pro^(3C^pro^H40A) added to translation-RNA replication reactions has a relatively small stimulating effect both on RNA synthesis and on the incorporation of [^35^S]-methionine into capsid precursors, empty capsids or particles sedimenting at 155S. These results are difficult to reconcile with the 100-fold increase in infectious virus observed in translation RNA-replication reactions that are supplemented with extra 3CD^pro^(3C^pro^H40A) [8,9]. Taken together these findings suggested the possibility that the presence of extra 3CD^pro^(3C^pro^H40A) enhances the specific infectivity of the virus particles produced, that is, it enhances the conversion of provirions to virions. To test this hypothesis we measured the yield of infectious virions in the peak fractions sedimenting at 155S in sucrose gradients derived from in vitro reactions incubated with or without extra 3CD^pro^(3C^pro^H40A). As shown on Fig. 5C, reactions to which extra 3CD^pro^(3C^pro^H40A) protein was added yielded 155S peaks containing 100 fold higher plaque forming units than reactions that were not supplemented with the protein. Interestingly, neither mutant 3CD^pro ^proteins (3C^pro^R84S/I86A or 3C^pro^H40G, 3D^pol^R455A/R456A) enhanced the virus yield in the 155S peak of the gradient (Fig. 5), an observation suggesting that both domains of the protein are required for this function. In a parallel experiment we have estimated the total number of viral particles in the 155S peak of the gradient by electron microscopy. We observed about 3-fold increase in viral particles when 3CD^pro^(3C^pro^H40A) was present in the translation-RNA replication reactions (data not shown).

To obtain further proof that the extra 3CD^pro^(3C^pro^H40A) enhances the specific infectivity of the virus particles we used SDS treatment of the reaction products prior to sucrose gradient analysis. The incorporation of [^35^S]-methionine into particles sedimenting at 80S and 155S was determined in reactions treated with SDS. It has been previously demonstrated that only mature virions but not provirions are stable in SDS [37]. As shown on Fig. 6A, there was no increase in 80S particles in SDS-treated samples that contained extra 3CD^pro^(3C^pro^H40A) (Fig. 6A) suggesting that the sample did not contain significant amounts of provirions. On the other hand, the 80S empty capsid peak, obtained from reactions with no extra 3CD^pro^(3C^pro^H40A) or with 3CD^pro^(3C^pro^H40G, 3D^pol^R455A/R456A) mutant protein, increased by about 4 fold as a result of SDS treatment. Interestingly, most of the extra label that appear in this 80S peak following SDS treatment is not derived from the 155S peak, presumably by the dissociation of provirions into 80S empty capsids and RNA (Fig. 6A). This suggested to us the possibility that in reactions lacking extra 3CD^pro^(3C^pro^H40A) some of the 80S particles aggregated and pelleted in the gradient. To test this possibility we recovered and analyzed the pellets from the gradients. We observed that the amount of [^35^S]-label in the pellet, derived from reactions with no extra 3CD^pro^, was 10-fold higher than in pellets of reactions lacking the extra protein (data not shown). A Western blot analysis of the particles in the pellets indicated the presence of VP0 but no VP2 (data not shown).

**Figure 7:**
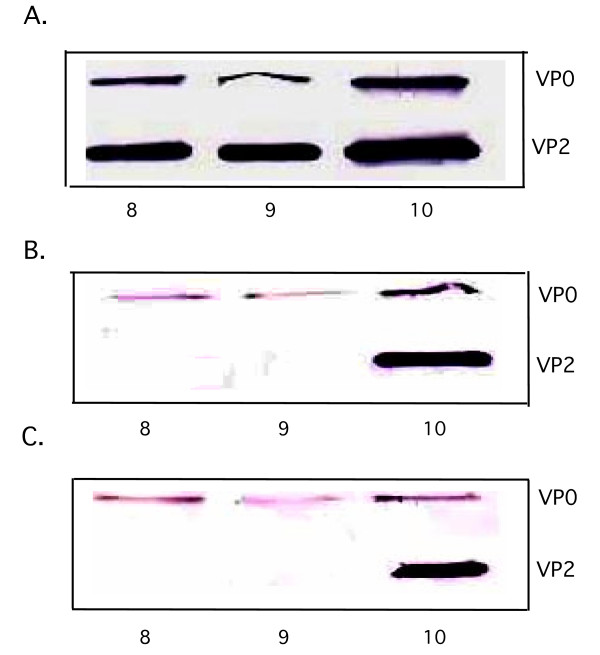
Comparison of the amount of VP0 and VP2 in 155S particles produced in reactions with and without extra 3CD^pro^(3C^pro^H40A). Translation RNA-replication reactions were carried out either in the absence or in the presence of extra 3CD^pro^(3C^pro^H40A) or 3CD^pro^(3C^pro^H40G, 3D^pol^R455A/R456A). The reaction products were separated on sucrose gradients, and the peak fractions were run on a SDS-polyacrylamide gel. Western blots were done with a polyclonal antibody to VP2 (Materials and Methods). The amount of VP0 and VP2 in fractions 8–10, in the 155S peak of the gradient shown on Fig. 6, was determined. (A) extra 3CD^pro^(3C^pro^H40A) added; (B) no extra 3CD^pro^(3C^pro^H40A) added; (C) 3CD^pro^(3C^pro^H40G, 3D^pol^R455A/R456A) added. Lane 1: fraction 8; lane 2, fraction 9; lane 3: fraction 10 of the 155S peak shown in Fig. 6.

As we discussed above, reactions containing extra 3CD^pro^(3C^pro^H40A) produced 3–7-fold higher amounts of 155S particles than those that lacked the extra protein (Figs. 5A,5B, 6). These particles were stable to SDS treatment (Fig. 6B) suggesting that they are mature virions. In contrast, the small peak of 155S particles obtained from reactions with no extra 3CD^pro^(3C^pro^H40A) or 3CD^pro^(3C^pro^H40G, 3D^pol^R455A/R456A) disappeared upon SDS treatment (Fig. 6B). These results suggest that under these conditions the 155S peaks consists of large amount of provirions that are dissociated into 80S particles and RNA by the SDS treatment. From the amount of [^35^S]-label resistant to SDS in the 155S peaks (Fig. 6) it can be estimated that the presence of extra 3CD^pro^(3C^pro^H40A) in translation-RNA replication reactions enhances the yield of mature virus about 40-fold. Western blot analyses with anti VP2 antibodies of gradient samples 8–9 from the 155S peak confirmed the presence of VP0, indicating provirions in reactions lacking extra 3CD^pro^(3C^pro^H40A) (Fig. 7B) or containing 3CD^pro^(3C^pro^H40G, 3D^pol^R455A/R456A) (Fig. 7C). Faster sedimenting particles in fraction 10 of this gradient contained some VP2 characteristic of mature virus. In contrast, reactions that contained extra 3CD^pro^(3C^pro^H40A) yielded a 155S peak containing predominantly VP2, as judged by the Western analysis (Fig. 7A). Therefore, we conclude that the extra 3CD^pro^(3C^pro^H40A) enhances the specific infectivity of the viral particles produced.

### 3CD^pro^(3C^pro^H40A) does not stimulate RNA synthesis or virus production in translation RNA-replication reactions programmed with transcript RNA

Transfection of full-length transcript RNAs of poliovirus, made by T7 RNA polymerase, into HeLa cells initiate a complete replication cycle although the yield of virus is only 5% of that obtained in transfections with virion RNA [38]. In the in vitro translation-RNA replication system the yield of virus with transcript RNAs is also significantly reduced to about 1% of what is obtained when the reactions are supplemented with viral RNA [39,40]. This has been attributed to the presence of two extra GMPs at the 5'-end of the transcript RNAs (pppGpGpUpU...), which are removed during replication to yield authentic viral RNA (VPg-pUpU...) [39]. Previous studies have demonstrated that the two GMPs at the 5' end of transcript RNAs do not interfere with minus strand RNA synthesis but greatly reduce the initiation of plus strand RNA synthesis in the in vitro system. Removal of the extra nucleotides with a *cis*-active hammerhead ribozyme resulted in templates that have regained most of their ability to support efficient plus strand RNA synthesis in the translation-RNA replication system [39].

**Figure 8:**
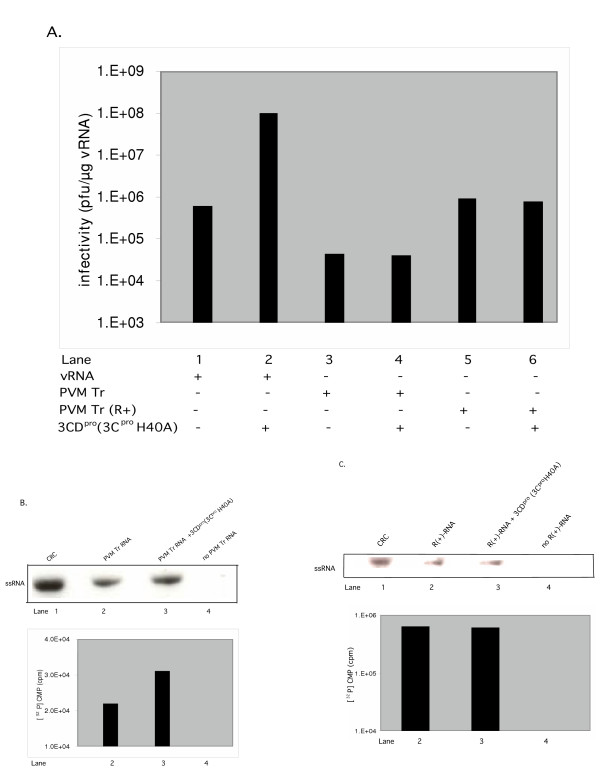
Extra 3CD^pro^(3C^pro^H40A) has no effect on virus production and RNA synthesis in reactions programmed with PV transcript RNA. Translation RNA-replication reactions were carried out, as described in Materials and Methods. The viral RNA template was replaced with a PV full-length transcript RNA made from a T7 promoter or with a ribozyme-treated transcript RNA. Where indicated 3CD^pro^(3C^pro^H40A) (5.5 nM) was added at t = 0 hr. (A) Comparison of virus yields in reactions templated with viral RNA and transcript RNAs. (B) Plus strand RNA synthesis with initiation complexes isolated from reactions programmed with PV transcript RNA made from a T7 promoter (Materials and Methods). Lane 1, CRC: [^32^P]-labeled RNA products obtained from crude replication complexes (Materials and Methods). (C) Plus strand RNA synthesis with initiation complexes isolated from reactions programmed with ribozyme-treated PV transcript RNA (R+ RNA). Lane 1, CRC: [^32^P]-labeled RNA products obtained from crude replication complexes (Materials and Methods).

To determine the effect of 3CD^pro^(3C^pro^H40A) on virus production, in reactions templated by transcript RNA, we have generated full-length PV transcript RNA from a T7 RNA polymerase promoter and used these to program in vitro translation-RNA replication reactions. In agreement with previous studies, we have observed that the virus yield is 50–100 fold lower in reactions programmed with transcript RNA instead of viral RNA (Fig. 8A, compare lane 1 with lane 3). In contrast, the yield of virus from reactions templated by ribozyme-treated transcript RNAs was essentially the same as what was obtained from viral RNA (Fig. 8A, compare lane 1 with lane 5). Remarkably, the virus yield was not enhanced by 3CD^pro^(3C^pro^H40A) in either reactions using transcript RNAs with or without ribozyme-treatment (Fig. 8A, compare lanes 5 and 6 and also lanes 3 and 4, respectively).

Previous studies have demonstrated that in the in vitro translation-RNA replication system the amount of plus strand RNA product obtained from PV ribozyme-treated transcript RNA or viral RNA is about 100-fold higher than what is produced in reactions with ribozyme-deficient transcript RNAs [40]. To examine whether the lack of enhancement of virus production by 3CD^pro^(3C^pro^H40A) in our reactions using a ribozyme-deficient transcript RNA is due to a defect in stimulating RNA synthesis we have measured the yield of plus strand RNA. Translation-RNA replication reactions were incubated for 4 hr at 34°C, the initiation complexes were collected by centrifugation and resuspended in reactions lacking transcript RNA. The RNA products were labeled with [α-^32^P]CTP for 1 hr and the products were applied to a nondenaturing gel. As shown in Fig. 8B, the presence of extra 3CD^pro^(3C^pro^H40A) in such reactions has no stimulatory effect on plus strand RNA synthesis (compare lane 2 with lane 3). As a size marker for plus strand RNA we have included the [α-^32^P]-labeled full-length PV ssRNA product made in CRCs (Fig. 8B, lane 1). The same results were obtained when RNA synthesis was measured with ribozyme-treated transcript RNA as template for translation-RNA replication (Fig. 8C, compare lane 2 with lane 3). It should be noted that the addition of extra 3CD^pro^(3C^pro^H40A) to translation reactions of transcript PV RNA had no effect either on the efficiency of translation or the processing of the polyprotein (data not shown).

### The lethal R84S/I86A mutation in the 3C^pro ^domain of 3CD^pro ^cannot be complemented in vitro by wt 3CD^pro^

It has been previously demonstrated that *in vivo *complementation rarely occurs, and if it does, it is very inefficient [7,41]. However, this process is more efficient in the in vitro system because large amounts of complementing proteins are translated from the input RNAs and these are apparently accessible to the replication complex [6]. Our results described in this paper indicate that at least 2 functions of 3CD^pro^(3C^pro^H40A) are complementable in the in vitro system and both of these functions depend on the RNA binding sequences of the protein. One of these is in RNA synthesis and the other one in virus maturation. To determine whether there are additional functions of 3C^pro^/3CD^pro ^that involve RNA binding we have attempted to complement the lethal R84S/I86A mutation in a full length PV transcript RNA either by cotranslation of wt 3CD^pro ^mRNA or by the addition of purified 3CD^pro^(3C^pro^H40A) to in vitro reactions. As shown in Table 1, the extra wt 3CD^pro ^does not restore the ability of the system to generate infectious virus. It should be noted that the 3CD^pro ^translated both from the mutant PV RNA and the 3CD^pro ^mRNA have full proteolytic activity (data not shown) and therefore these results are not due to a defect in protein processing. We have obtained the same negative results when we cotransfected the R84S/I86A mutant full length PV RNA with wt 3CD^pro ^mRNA into HeLa cells (data not shown). These results can be interpreted to mean that: (1) 3CD^pro ^has one or more additional RNA binding function(s), which is not complementable; (2) that an RNA binding function of 3C^pro ^cannot be complemented by 3CD^pro^.

**Table 1:** Mutation R84S/I86A in the RNA binding domain of 3C^pro ^cannot be complemented in vitro with wt 3CD^pro^.^a^

Sample	Infectivity (pfu/μg transcript RNA)
PVM(3C^pro^R84S/I86A) Tr RNA	0
PVM(3C^pro^R84S/I86A) Tr RNA + 1.4 μg/ml 3CD^pro ^mRNA	0
PVM (3C^pro^R84S/I86A) Tr RNA + 400 ng/ml 3CD^pro ^protein	0

^a ^Translation RNA-replication reactions were carried out with a PVM transcript RNA, containing the R84S/I86A mutations in 3C^pro ^as template. Where indicated the reactions were supplemented with wt 3CD^pro ^mRNA (1.4 μg/ml) or 3CD^pro^(3C^pro^H40A) purified protein (5.5 nM). The virus yield was measured with a plaque assay (Materials and Methods).

## Discussion

We have previously shown that the level of active 3CD^pro ^in in vitro translation-RNA replication reactions, programmed with viral RNA, is suboptimal for efficient virus synthesis and that the addition of extra 3CD^pro ^compensates to some extent for this deficiency [8,9] but the reason for this phenomenon remained unsolved. The results presented in this paper indicate that the stimulatory effect of 3CD^pro ^is both at the level of RNA synthesis and of virus maturation. Since translation, replication, and encapsidation are coupled processes during the growth of poliovirus [13,42,43] one might conclude that the increase in the yield of mature virions simply reflects the stimulation of RNA synthesis. However, although this might be true to some extent, our results indicate that 3CD^pro^(3C^pro^H40A) exerts its enhancing activity at two distinct stages of the viral growth cycle. This conclusion is supported by three lines of evidence: (1) plus strand RNA synthesis is stimulated by 3CD^pro^(3C^pro^H40A) about 3-fold but the yield of progeny virus increases 100 fold; (2) although 3CD^pro^(3C^pro^H40G, 3D^pol^R455A/R456A), containing mutations at interface I in the 3D^pol ^domain of the protein, enhance RNA synthesis nearly as efficiently as 3CD^pro^(3C^pro^H40A) it does not stimulate the yield of mature virus; (3) only those reactions that contain extra 3CD^pro^(3C^pro^H40A) yield a 155S peak in sucrose gradients with particles resistant to SDS treatment.

Our results with the in vitro translation-RNA replication system do not define the precise role of the extra 3CD^pro ^in stimulating RNA synthesis. The evidence available thus far indicates that in the presence of extra 3CD^pro^(3C^pro^H40A) (1) minus and plus strand RNA synthesis are stimulated 2- and 3-fold, respectively; (2) the RNA binding sequences (R84/I86) in the 3C^pro ^domain of the polyprotein are required for the stimulation; (3) the integrity of interface I in the 3D^pol ^domain of the polyprotein is not important. Whether plus strand RNA synthesis itself is stimulated by the presence of extra 3CD^pro ^or the amount of plus strands increases simply as a result of more minus strands remains to be determined. The fact that the RNA binding domain of the protein in 3C^pro ^is involved in stimulating RNA synthesis suggests that the extra 3CD^pro ^forms a functional ribonucleoprotein complex (RNP) with an RNA sequence or structure in the viral genome. Poliovirus RNA contains at least 3 different *cis*-acting elements that are involved in RNA replication. All of these bind 3CD^pro^, the 5' cloverleaf [17,18,22], the *cre*(2C) element [20,21] and the 3'NTR [19]. From these 3 structures only the 5' cloverleaf [18,19,22,44] and the *cre*(2C) stem loop structure [20,21,45] have been shown so far to form a biological relevant RNP complex with 3CD^pro^. The cloverleaf has been shown to be required for minus strand, and possibly also for plus strand RNA synthesis [17,46]. The RNP complexes of the cloverleaf with 3CD^pro^, which also include either PCBP2 or 3AB, are also required for both minus and plus strand RNA synthesis [17,19,44,47].

The other important *cis*-replicating element involved in poliovirus RNA replication, which also binds 3CD^pro^, is the *cre*(2C) hairpin [20,21,45]. A conserved AAA sequence in this RNA element serves as template for the synthesis of VPgpU(pU), the primer for RNA synthesis [20,45]. The role of 3CD^pro ^in this reaction is believed to be to enhance the binding of the polymerase/VPg complex to the *cre*(2C) element [20,21,45]. The question whether the VPgpU(pU) made in this reaction is used exclusively for plus strand RNA synthesis [4] or also for minus strand synthesis remains controversial. The RNA binding sequences (R84/I86) of 3C^pro ^in 3CD^pro ^but not amino acids R455/R456 at interface I in the 3D^pol ^domain are essential for the protein's stimulatory activity both in VPg-uridylylation in vitro [20,33] and in the stimulation of RNA synthesis in the translation-RNA replication system. Taken together these results are consistent with a possible role of either the 3CD^pro^/cloverleaf or the 3CD^pro^/*cre*(2C) interactions in the stimulatory activity of the protein in RNA synthesis, which is dependent on the RNA binding activity of the 3C^pro ^domain.

We have previously reported the interesting observation that the addition of purified protein 3C^pro^(C147G) along with 3CD^pro^(3C^pro^H40A) to translation-RNA replication reactions reduces the virus yield about ten thousand fold [8]. In this paper we show that at least one of the reasons for the nearly total inhibition of virus production under these conditions is that there is a striking inhibition of both minus and plus strand RNA synthesis. One possible explanation of our in vitro results is that the two proteins form a complex, through intermolecular contacts in 3C^pro ^[48], which is inactive and either cannot bind to the RNA or the RNP complex is nonfunctional. Alternatively, the two proteins interact with the same RNA sequence or structure but only the 3CD^pro^/RNA complex is functional in RNA synthesis. Of the three *cis*-replicating elements contained within PV RNA both the cloverleaf and the *cre*(2C) element have been shown to form RNP complexes with either 3CD^pro ^or 3C^pro ^[17,21]. In case of the cloverleaf only the 3CD^pro^/RNP complex is functional in replication but both protein-RNA complexes stimulate VPg-uridylylation on the *cre*(2C) RNA element [33]. These results suggest that the RNA sequence or structure involved in the stimulatory activity of 3CD^pro ^in RNA synthesis in the in vitro system is the cloverleaf rather than the *cre*(2C) element.

As we discussed above, the second step in the life cycle of PV where the extra 3CD^pro^(3C^pro^H40A) appears to exert its stimulatory effect in vitro is during the late stages of particle assembly, and in particular during virus maturation. Although the addition of extra 3CD^pro^(3C^pro^H40A) leads to a slight increase in the amount of small capsid precursors, the primary effect of the protein is at the step during which provirions are converted to mature viral particles. Although the mechanism of maturation cleavage is not fully understood it has been well established that the process is dependent on the presence of viral RNA [reviewed in [49]]. The exact function of 3CD^pro^(3C^pro^H40A) in virus maturation is not yet known. Interestingly, both the RNA binding sequences in 3C^pro ^and the integrity of interface I in the 3D^pol ^domain of 3CD^pro ^are required for function but the proteolytic activity of the protein is dispensable. The fact that the RNA binding domain of 3C^pro ^is essential for function indicates that 3CD^pro ^has to interact with a sequence or structure in the viral RNA. The observation that the integrity of interface I in the 3D^pol ^domain of the protein is also required for this process is more difficult to explain. Although the oligomerization of 3CD^pro ^along interface I in 3D^pol ^has not yet been directly tested, recent structural studies of the RNA polymerase suggest that oligomerization of the protein along interface I is possible [30]. In addition, recent studies of genetically modified 3CD^pro ^polypeptides in RNA replication strongly support a role of 3CD^pro^/3CD^pro ^complexes, mediated by 3D^pol ^domain contacts [50]. Whether the function of interface I in the 3D^pol ^domain of 3CD^pro ^in virus maturation is related to the RNA binding properties of the protein remains to be determined. Our recent in vitro studies indicate that mutation 3D^pol^R455A/R456A in the context of 3CD^pro ^alter the RNA binding properties of the protein such that twice as much of the mutant protein is required for optimal binding to a *cre*(2C) RNA probe than of the wt protein [Pathak and Cameron, unpublished results]. Oligomerization of 3CD^pro ^might also be aided by intermolecular contacts between the 3C^pro ^domains of two molecules [48]. However, it should be noted that no interaction can be detected between 3C^pro ^molecules in chemical cross-linking experiments in vitro and only very poor, if any, complex formation can be observed between either 3C^pro^/3C^pro ^or 3CD^pro^/3CD^pro ^molecules in the yeast two hybrid system [51].

On the basis of these observations we propose 2 possible models for efficient virus maturation in the in vitro translation-RNA replication reactions supplemented with extra 3CD^pro^(3C^pro^H40A). According to the first model 3CD^pro^(3C^pro^H40A) interacts with the progeny plus strand RNA, possibly at the cloverleaf, and causes an important conformational change. This step requires the RNA binding activity of the 3C^pro ^domain of the protein but binding might also be enhanced by the oligomerization of the polypeptide along interface I in the 3D^pol ^domain. Subsequently the RNA interacts either with the pentamers or the empty capsid and it is encapsidated, yielding a provirion while 3CD^pro^(3C^pro^H40A) leaves the complex. The correct conformation of the RNA inside the provirions affects the shape of the capsid such that now the cleavage of the VP0s is favored to complete maturation. The second model is similar to the first one except that now 3CD^pro^(3C^pro^H40A) itself is encapsidated, bound to the progeny RNA. This keeps the RNA in the correct conformation inside the capsid so that the maturation cleavage of VP0 can occur. The second model is supported by previous studies by Newman and Brown who observed that 3CD^pro^, 3D^pol ^and 2C^ATPase ^proteins were contained within isolated poliovirus and foot-and-mouth disease virus particles [52]. In this context one should note that the scissile bond in VP0 is located on the rim of a trefoil-shaped depression on the capsid's inner surface, which has the potential of binding either RNA or other macromolecules [11]. However, we did not detect any 3CD^pro ^in our 155S peak derived from reactions with extra 3CD^pro^(3C^pro^H40A) using Western blot analysis with either anti 3C^pro ^or anti 3D^pol ^antibodies [data not shown]. In any case, the suboptimal concentration of functional 3CD^pro ^in translation RNA-replication reactions might lead to progeny RNA molecules lacking the proper conformation for encapsidation and efficient virus maturation.

One of the factors that limits the use of the in vitro translation-RNA replication system in studies of RNA replication is the poor function of transcript RNAs as templates in the reaction, lowering the yield of progeny plus strand RNA and of virus to about 1% of what is obtained with virion RNA [39,40]. This has been attributed to the presence of two GMP molecules at the 5' end of RNAs transcribed from a T7 promoter [39]. We hoped that by supplying the inefficient in vitro reactions with an excess of 3CD^pro^(3C^pro^H40A) the synthesis of plus strands, and consequently the production of mature virus could be enhanced. To our surprise, this does not happen. The simplest explanation of these observations is that the level of endogenous 3CD^pro ^is sufficient for the synthesis of the low level of plus strand RNA that is produced in the system. Therefore supplying the reactions with extra 3CD^pro^(3C^pro^H40A) would have no stimulatory effect. However, this explanation does not account for the fact that virus synthesis is not stimulated by 3CD^pro^(3C^pro^H40A) in reactions containing ribozyme-treated transcript RNAs. The yield of virus in such reactions is 50-fold higher than in samples in which ribozyme-deficient transcripts were used as template for translation and RNA replication. The only known difference between viral RNA and ribozyme-treated transcript RNA is the lack of VPg in the latter structure. Therefore our results indicate that the presence of VPg at the 5' end of the input viral RNA [53,54] is an important determinant of the ability of 3CD^pro^(3C^pro^H40A) to stimulate RNA synthesis and production of viable virions. Interestingly, the addition of extra 3CD^pro^(3C^pro^H40A) at the beginning of incubation does not stimulate these processes once the newly made VPg-linked viral RNAs are used as templates for replication and packaging. This suggests that at least one of the stimulatory functions of 3CD^pro ^is required at the time RNA synthesis is initiated from the input VPg-linked RNA template. Our results also suggest that either directly or indirectly the presence of VPg on the input RNA template is important for the stimulation by 3CD^pro^(3C^pro^H40A) of the encapsidation of the newly made viral RNAs. The involvement of VPg in encapsidation has been previously proposed by Reuer et al. [15] who observed that some lethal VPg mutations still permit near normal minus and plus strand RNA synthesis in vivo.

It has been known for some time that complementation between viral proteins is more efficient in the in vitro translation-RNA replication system than in vivo. This is most likely due to relatively large local concentrations of viral proteins that are translated from the input viral RNA template used in the in vitro reactions. The results described in this paper show that at least two functions of 3CD^pro ^are complementable in vitro. One is in RNA synthesis and the other in virus maturation and both of these processes require the RNA binding sequence of the 3C^pro ^domain. In an attempt to determine whether the RNA binding function of 3CD^pro^(3C^pro^H40A) is required for additional processes in viral growth we tried to complement the lethal 3C^pro^R84S/I86A mutation in the PV genome in vitro either by the addition of 3CD^pro^(3C^pro^H40A) protein or wt 3CD^pro ^mRNA. We obtained no virus suggesting that one or more of the RNA binding functions of 3CD^pro^, distinct from the ones described by us, cannot be complemented in vitro. An alternate explanation of the observation is that 3CD^pro ^cannot substitute for 3C^pro ^in one or more of its functions.

The results presented in this paper have yielded insights into the steps of the viral life cycle in which the extra 3CD^pro^(3C^pro^H40A) exerts its stimulatory function in the translation-RNA replication system. Our results also suggest a new role for protein 3CD^pro ^in the life cycle of poliovirus, in virus maturation, which is dependent on the integrity of interface I in the 3D^pol ^domain of the protein. In addition, we have shown that a VPg-linked PV RNA linked template and the 3C^pro ^domain of the 3CD^pro^(3C^pro^H40A) polypeptide are required both for the stimulation of RNA synthesis and for virus maturation. However, the exact mechanism of stimulation by 3CD^pro ^both during RNA synthesis and particle assembly remains to be determined.

## Materials and methods

### Cells and viruses

HeLa R19 cell monolayers and suspension cultures of HeLa S3 cells were maintained in DMEM supplemented with 5% fetal bovine calf serum. Poliovirus was amplified on HeLa R19 cells as described before. The infectivity of virus stocks was determined by plaque assays on HeLa R19 monolayers, as described before [55].

### Preparation of poliovirus RNA

Virus stocks were grown and purified by CsCl gradient centrifugation [55]. Viral RNA was isolated from the purified virus stocks with a 1:1 mixture of phenol and chloroform. The purified RNA was precipitated by the addition of 2 volumes of ethanol.

### Preparation of HeLa cytoplamic extracts

HeLa S10 extracts were prepared as previously described [1,56] except for the following modifications: (1) packed cells from 2 liters of HeLa S10 were resuspended in 1.0 volumes (relative to packed cell volume) of hypotonic buffer; (2) the final extracts were not dialyzed.

### Translation-RNA replication reactions with HeLa cell-free extracts and plaque assays

Viral RNA was translated at 34°C in the presence of unlabeled methionine, 200 μM each CTP, GTP, UTP, and 1 mM ATP in a total volume of 25 μl [1,5]. After incubation for 12–15 hr the samples were diluted with phosphate-buffered saline and were added to HeLa cell monolayers. Virus titers were determined by plaque assay, as described previously [1,55].

### Filter binding assays for measurement of total RNA synthesis

Method I. Translation-RNA replication reactions (125 μl) were incubated at 34°C in the presence of 62.5 μC of [α-^35^S]CTP (ICN, 600Ci/mmole) but lacking unlabeled CTP. At the indicated times samples were taken and the reactions were stopped by the addition of SDS to a final concentration of 0.5%. The samples were extracted with phenol-chloroform and the RNA was precipitated with ethanol. The pellets were resuspended in 10 mM Tris pH 7.5, 1 mM EDTA and were loaded on a DEAE-81 filter papers (Whatman). The filters were dried and subsequently washed three times with 5% Na_2_HPO_4_, once with water and once with 70% ethanol, as described before [57]. Method II. Each translation-RNAreplication reaction was incubated separately at 34°C. At the indicated times (2, 4, 6, 8, and 16 hr) 12.5 μC of [α-^35^S]CTP was added and incubation was continued for 1 hr. The samples were treated and analyzed as described in Method I.

### Preinitiation RNA replication complexes

Preinitiation RNA replication complexes were prepared as described previously [34] except for some minor modifications. Translation-RNA replication reactions, lacking initiation factors, were incubated for 4 hr at 34°C either in the presence or absence of 2 mM guanidine HCl. The complexes were isolated by centrifugation, resuspended in 50 μl HeLa S10 translation/replication reaction mixture without viral RNA, and incubated for 11 hr at 34°C.

### Plus and minus strand RNA synthesis

Plus and minus strand RNA synthesis were determined as described previously [2]. Translation RNA replication reactions, programmed with viral RNA, were incubated for 4 hr in the presence of 2 mM guanidine HCl. The preinitiation replication complexes were resuspended in translation-RNA replication reactions lacking viral RNA in the presence of [α-^32^P]CTP. The reactions were incubated at 34°C for 1 hr, the labeled RNAs were separated by native agarose gel electrophoresis, and the products were visualized by autoradiography. The reaction products were quantitated with a Phosphorimager (Molecular Dynamics Storm 800) by measuring the amount the amount of [α-^32^P]CMP incorporated into RNA.

Alternatively, plus strand RNA synthesis was measured in translation-RNA replication reactions that were incubated for 4 hr at 34°C, in the absence of guanidine HCl, and the initiation complexes were isolated by centrifugation. They were resuspended in translation-RNA replication reactions lacking viral RNA but supplemented with [α-^32^P]CTP. The samples were incubated for 1 hr at 34°C and the RNA products were separated on a native agarose gel. The products were visualized by autoradiography.

### In vitro transcription and translation

All plasmids were linearized with *EcoR*I prior to transcription by T7 RNA polymerase. The transcript RNAs were purified by phenol/chloroform extraction and ethanol precipitation. Translation reactions (25 μl) containing 8.8 μC of Trans [^35^S]Label (ICN Biochemicals) were incubated for 4 hours at 34°C [5]. The samples were analyzed by electrophoresis on sodium deodecyl sulfate-12% polyacrylamide gels, followed by autoradiography.

### RNA synthesis with crude replication complexes

Crude replication complexes (CRCs) were prepared by a method similar to what has been described before [35]. HeLa cell monolayers (15 cm) were infected with PVM at a multiplicity of infection of 500. After 6 hr incubation at 37°C the cells were resuspendend in hypotonic buffer [35] and were lysed with a Dounce homogenizer. Cell debris and nuclei were removed by centrifugation for 20 min at 33,000 × g. The pellet was subsequently resuspended in 1 ml of 10 mM Tris-HCl pH 8.0, 10 mM NaCl, and 15% glycerol. Aliquots were stored at -80°C.

RNA synthesis by CRCs was measured as described before [3]. In vitro translation-RNA replication reactions were assembled in which the HeLa extracts were replaced by CRCs (20% by volume). The reaction contained 49% by volume of S10 buffer [2] and 25 μC of [α-^32^P]CTP.

### Sucrose gradient centrifugation of viral particles

HeLa S10 translation-RNA replication reactions (25 μl) were incubated in the presence of 8.8 μC of [^35^S]TransLabel (ICN Biochemicals) for 12 hr at 34°C. The excess unincorporated label was removed by dialysis. The samples were introduced into a Slide-a-lyzer (Pierce Endogen) dialysis cassette with a M.Wt cut-off of 10 kD and were dialyzed several times against phosphate buffer at 4°C until essentially all the excess label was eliminated. After dialysis the samples were centrifuged at 14,000 × g to remove any precipitated material. The samples were diluted to 500 μl and were centrifuged in a 5–20% sucrose density gradient in phosphate buffered saline containing 0.01% bovine serum albumin in a SW41 rotor at 40,000 rpm at 4°C. To separate 80S empty capsids and 155S virus particles (provirions and virions) the gradients were centrifuged for 80 min [36]. To identify 5S protomers and 14S pentamers the gradients were centrifuged for 15 hr. Fractions (0.5 ml) were collected from the bottom of the gradients and the radioactivity of each sample was determined by scintillation counting. In each sucrose gradient cetrifugation size markers were sedimented in parallel consisting of [^35^S]-labeled PV-infected HeLa cell extracts.

### Western blot analysis

For the identification of the capsid proteins present in sucrose gradient fractions Western blot analysis was used [58]. Samples were loaded on a SDS-polyacrylamide gel (12.5% acrylamide) and after separation the proteins were transferred to a nitrocellulose membrane (Protran; Schleicher&Schuell). The membrane was probed with a rabbit polyclonal antibody to PV capsid protein VP2.

### Electron microscopy

Standard electron microscopy processing techniques were used for negative staining. Briefly, formvar coated, 200 mesh nickel grids were prepared. Grids, sample side down were floated on droplets of suspended poliovirus, followed by fixation in a solution of 1% glutaraldehyde in 0.1 M phosphate buffered saline (PBS), pH 7.4. Samples were washed in PBS, then in water followed by phosphotungstic acid. The samples were viewed with a F. E. I. Tecnai 12 BioTwin electron microscope and digital images were captured with an ATM camera system. In each sample the viral particles were counted within a 20 mm^2 ^area.

### Proteins

The following PV proteins with a C-terminal his tag were expressed in *E. coli *and purified by nickel column chromatography (Qiagen): 3CD^pro^(3C^pro^H40A), a proteinase active site mutant [20]; 3C^pro^(3C^pro^C147G), a proteinase active site mutant [33]. The purification of 3CD^pro^(3C^pro^H40G, 3D^pol^R455A/R456A) was described previously [33]. This protein contains both a proteinase active site mutation (3C^pro^H40G) and a mutation (3D^pol^R455A/R456A) at interface I in the 3D^pol ^domain of the protein.

### Plasmids

Poliovirus sequences were derived from plasmid pT7PVM, which contains the full-length (nt 1–7525) plus strand poliovirus cDNA sequence [38]. All constructs were sequenced to ensure their accuracy. The construction of plasmids pLOP315ser and pLOP315(3C^pro^R84S/I86A) was described before [8,9]. Both plasmid DNAs were linearized with Eco*RI *prior to transcription with T7 RNA polymerase.

## Authors' contributions

DF carried out all the experiments and made substantial contributions to the design of the experiments. HP contributed purified mutant enzymes for the study. CEC has contributed to the interpretation of the data and revised the manuscript critically. BR initiated the studies on this subject. EW contributed to the design of the experiments and revised the manuscript critically. AVP planned the experiments and wrote the manuscript. All authors read and approved the final manuscript.
